# Equal Alternatives or Lower Standards for Immigrant Women—Analyzing Obstetric Care for Immigrant Women in Berlin Within the Framework of Cultural Health Capital

**DOI:** 10.1007/s40615-023-01732-0

**Published:** 2023-08-15

**Authors:** Claudia Großkreutz, Burcu Gürbüz, Theda Borde, Rebecca C. Rancourt, Wolfgang Henrich, Matthias David, Vera Seidel

**Affiliations:** 1 Clinic of Obstetrics, Charité-Universitätsmedizin Berlin, Campus Virchow-Klinikum, Berlin, Germany; 2 Alice Salomon Hochschule, Berlin, Germany; 3 Division of ‘Experimental Obstetrics’, Clinic of Obstetrics, Charité-Universitätsmedizin Berlin, Campus Virchow-Klinikum, Berlin, Germany; 4 Clinic of Gynecology, Charité-Universitätsmedizin Berlin, Campus Virchow-Klinikum, Berlin, Germany

**Keywords:** Cultural Competency, Immigrant health, Culture, Healthcare Disparities, Professional-Patient Relations

## Abstract

In obstetric clinics in Berlin, Germany, more than half of the women are immigrants. The main objective of the qualitative study was to explore the staff’s experiences with obstetric care for immigrants and juxtapose it with the immigrants’ comments on their birth experiences. We analyze potential differences along the framework of a cultural health capital (CHC). Between May and August 2017, semi-structured interviews were carried out with 17 obstetricians and 17 midwives at four obstetric clinics in Berlin. The verbally transcribed interview material was subjected to a qualitative content analysis according to Mayring. Furthermore, a secondary data from an interview study was analyzed in the purpose of providing some insight into the practitioner study participant perspective. Between January and May 2017, in the postpartum ward at the Berlin Charité Campus Virchow Clinic, an interview study guided by the migrant-friendly maternity care questionnaire was conducted among 410 migrant and non-migrant women. For this study, the free-text comments on the pregnancy care were analyzed. The staff interviewees identified language barrier and legal status as risk factors for the late onset of obstetric care. CHC functioning potentially as alternatives to the established health care structures were voiced. Strong family ties among immigrant families bear a high potential for support. Gratefulness was voiced by the staff and immigrant patients as a source of satisfaction with care. Our study shows that obstetric care for immigrant women remains a challenge. CHC of immigrant women might partially compensate for exclusion.

**Supplementary Information:**

The online version contains supplementary material available at 10.1007/s40615-023-01732-0.

## Introduction

Migration has long been a consistent global phenomenon; however, a rising tendency has been observed over the past few years. Germany has approximately 82 million inhabitants, of which according to the latest population-based survey from 2019, 16.8% have been born abroad and migrated to Germany and another 9.2% have grown up in Germany but have parents that have migrated to Germany [1].

It follows from these recent trends that health care professionals providing obstetric care are in a more frequent contact with immigrant women. Care for refugee women can be challenging especially regarding communication barriers, living situation of refugee women, psychological distress after history of flight, and unmonitored pregnancies [2, 3]. We showed in a recent study that 21% of immigrant women compared to 11% of non-immigrant women started prenatal care late, i.e. beyond the first trimester. Furthermore, a greater proportion of immigrant women (23%) than non-immigrant women (3%) were less informed on the availability of institutionalized postpartum care as well as postpartum midwifery care [4]. Another recent study by our study group on perceptions of midwives and doctors on obstetric care for immigrant women shows that especially in the case of a language barrier medical personal feels stressed and worries about quality of care [5].

Internationally, health care providers’ perception on providing pregnancy and obstetric care to immigrant women has been studied and challenges have been described. A study in English NHS maternity units showed that staff found it easier to provide care to UK-born women as opposed to immigrant women [6]. A study from the Netherlands highlighted how midwives perceived it as demanding but rewarding providing care to immigrant women [7]. When being interviewed about their experiences in providing health care to non-western women, midwives in the Netherlands mentioned a greater difficulty in performing their job due to communication problems, lack of knowledge of the health care system on behalf of the patients, and cultural-based beliefs of family members especially in postpartum care [7]. A Swedish study investigated factors influencing maternity care which seems to be unequal to the native women, finding good communication to be very important as much as building a trustful relationship with the women [8]. However, Canadian researchers indicated differing values between immigrant women and the health care system where the women’s specific needs should be considered more [9]. The only study on this topic in Germany is by Anne Kasper but focused only on refugee women [10].

### Cultural Health Capital (CHC)

To investigate how immigrant women might partially be excluded from the host countries’ health care system, it is helpful to investigate the underlying reasons and dynamics. A theoretical framework to understand how health resources and subsequently outcomes are unequally distributed in the health care system is the concept of cultural health capital (CHC). CHC is usually used to describe how ethnic minorities integrate into the dominant health care system. Combined with critical race theory it has been used to show how immigrants make use of their cultural health capital in a way outside of the dominant health care system. Hence, in this paper, we used the concept on how Erin F. Madden [11] combined the conceptual framework of CHC by Janet K. Shim [12] and the critical race theory by Tara J. Yosso [13]. Pierre Bourdieu explained the inequalities in society through differently allocated “resources” or “capital”. He distinguished between “economic capital” such as income and property, “social capital” meaning access to relationships and networks, and “cultural capital” describing skills and knowledge of a person [14]. Shim developed her theoretical framework grounded in Bourdieu’s notion of cultural capital and made it usable for analysis of interactions in the health care system [12]. Shim defined in her conceptual article CHC as “the repertoire of cultural skills, verbal and nonverbal competencies, attitudes and behaviors, and interactional styles, cultivated by patients and clinicians alike, that when deployed, may result in a more optimal health care relationships.” Shim et al. showed through a qualitative study in the western US how CHC can facilitate the entry to the dominant health care system [15]. In their qualitative study conducted between 2010 and 2011 they showed how good communication between provider and patient is more likely, when the patient expresses his or her symptoms and needs in a way that the provider expects him or her to do so. They highlight the power that the provider has in terms of defining which kind of behavior leads to subsequent actions. The more CHC a patient has of the dominant provider’s culture, the more likely it is that he received genuinely patient-centered care. The concept has also been used to assess the attitudes and experiences of an intervention trial in the Danish antenatal care system among midwives and immigrant women [16]. Yosso criticized Bourdieu’s concept of cultural capital with the critical race theory. According to her, cultural capital as an entry to the dominant system, in her example the education system, focusses too much on deficits [13]. In her work, she focusses on the cultural resources that marginalized groups have. According to Yosso, it is necessary that schools are tailored to the respective communities they serve. In her example, she calls for the development of schools that acknowledge the numerous strengths of African American students. For the analysis of intercultural interaction in the health care system, Madden brought the concept of CHC and critical race theory together. She highlights which resources a cultural community mobilizes to respond to exclusion from the dominant health care system. In her work on Mexican American communities, she showed that CHC cannot overcome the exclusion from the dominant health care system, but that it helps to find an alternative system of access to doctors and prescription drugs [11]. She found that while for some patients, prescriptions are too expensive to redeem in the USA so they illegally import them from Mexico, where they can be bought at a cheaper price. Madden’s work highlights how exclusion from the dominant health care system can lead to the creation of alternative structures that mitigate the effects of exclusion. She stresses that these alternatives are often not equal in quality of care, as for example the illicit purchase of drugs can lead to improper dosage. It is none the less crucial for providers to know these alternative structures built up through CHC to react to them [11].

In our qualitative study on the health care staff’s perception of obstetric care in Berlin, differences between immigrant and non-immigrant women were expressed by the respondents. In this paper, we briefly describe these observed differences. The analysis in this paper focuses on immigrant women’s resources observed by midwives and obstetricians working in Berlin with the help of the concept of CHC. The research question in this paper is what CHC immigrant women are resorting to in Berlin during pregnancy and birth. We will argue that CHC presented by Madden—understood as alternatives to the host countries’ health care system—can be seen as a resource, but we will highlight that common standards have to be defined to avoid ambivalences in judgement of differences.

## Materials and Methods

Between May and August 2017, 34 semi-structured oral face-to-face interviews with obstetricians and midwives delivering peripartum care were conducted by a research team that consisted of two medical students and one physician working in obstetrics and gynecology (all women). These interviewers had no prior contact or relation to the participants and were all previously trained in qualitative data acquisition. Ethics approval from the ethics committee of the Charité-Universitätsmedizin Berlin (EA2/187/16) and consent from the respective employee representing bodies of the participating clinics were obtained. The respective heads of the obstetric departments were contacted by the study group via email. Four clinics with more than 3000 births per year and all level one perinatology centers (centers of highest standard of care in perinatology in Germany) were purposely chosen for qualitative interviews, two in the former Eastern (German Democratic Republic) and two in the former western part of Berlin (Federal Republic of Germany). The female immigrant population in each district in the year of the study (2017) was composed a little bit differently, but approximately 1/3 of immigrants (23–32%) were from the EU, with Poland being the dominant country of origin. In three districts, the main regions of origin outside the EU were Islamic countries including Turkey and Syria (40–32%). The second region of origin most often encountered were countries of the former Soviet Union (11–6%). The fourth district had mostly immigrants from both regions just in different proportions: 29% former Soviet countries and 26% Islamic countries[17]. As pregnant women have a free choice for their hospital, the immigrant composition of the respective district does not necessarily exactly reflect the composition of immigrant patients in each hospital. In all four clinics, an equal number of midwives and obstetricians have been interviewed. The participation was open to every midwife and obstetrician actively attending births. Midwifery students or obstetricians who were not actively attending births were excluded. After oral and written information about the study, the potential participants decided against or for participation. Written consent was obtained before the start of the interview. Prior to the start of each interview, a questionnaire on sociodemographic characteristics was filled out by the participants. The semi-structured interviews were based on an interview guideline and covered various predefined issues (see appendix 1). The term “immigrant” was defined during the interview as a person with personal experience of migration. The interviews were audiotaped and subsequently verbally transcribed. To ensure confidentiality, all information allowing any identification of the person or corresponding clinic was removed from the transcripts. Transcripts were checked twice (by the first and last author) to ensure the accuracy of the transcription before coding. Qualitative content analysis was performed inductively according to the steps proposed by Mayring [18]. Coding was performed by two researchers (first and last author). Data analysis was initiated parallel to the conduction of interviews so that data saturation could be detected during the period of data collection [19] and the research could accordingly be concluded after 34 of the initial 40 planned interviews. Analytical codes were organized into main themes and subthemes with the program MAXQDA Plus 12 (Release 12.3.1, VERBI GmbH Berlin). Coherence of coding was checked in a random sample of interview transcripts and discrepancies were discussed between the researchers to ensure the validity and the credibility of the results.

Between January and May 2017 in the postpartum ward at the Charité Campus Virchow-Clinic, Berlin, an interview study guided by the migrant-friendly maternity care questionnaire [20] was conducted. For details about the quantitative analysis of this study see previous publications [4, 21]. The 410 migrant and non-migrant women participating in this study also had the opportunity and were encouraged to add free-text comments on the pregnancy care they received in Germany. For this study, the secondary data was analyzed in the purpose of providing some insight into the practitioner study participant perspective. The additional comments from immigrant women gathered in the 2017 study were used to consider the themes expressed by the practitioner participants in this study.

## Results

Five midwives and five obstetricians were interviewed in three hospitals each and two obstetricians and two midwives in the fourth hospital. Of the total 34 participants, 30 were women and four were men with equally distribution of 17 midwives and 17 obstetricians. A total of 12 h and 15 min of recorded interview material was analyzed. Redundancy already occurred after 23 interviews; however, the total reached 34 as dates with the participants had already been scheduled.

The median age of participants was 35.5 years (range: 22–56 years) with a median of 6 years of work-experience (range: 1 month–32 years). Three participants were themselves immigrants. All participants born in Germany were German native speakers. Three of the German native speakers had an additional second native language. Three participants had another native language than German. Native languages other than German were English, Spanish, Turkish, and Arabic. All participants spoke at least one foreign language including English, French, Spanish, German, Russian, Polish, Greek, or Italian.

The way midwives and obstetricians identified immigrant women was foremost by their language proficiency as well as their names.

*“Well, often it is straightforward as they do not really speak German.”* (Physician, 43 years old)

*“Well the communication as first probably.”* (Midwife, 28 years old)

*“by the name, of course, that is the first thing we see, before we even see the patient.”* (Physician, 36 years old)

*“when I look at the name, I honestly have to admit, I am always a little bit relieved when I read a German name.”* (Physician, 28 years old)

Some interviewees also voiced physical appearance, clothes, health insurance, or cultural habits as a way they identified immigrant women.

*“for once the language. When there is a language barrier, that is the most evident actually. And also the appearance of course. These are the most important.”* (Midwife, 39 years old)

*“hm, often language skills and if they know how the system works. Also, often it is clear because they don’t really speak German. […] they have a different health insurance because they are asylum seekers or they tell me as well.”* (Physician, 43 years old)

*“of course, there are certain things. For example, German women often come with their husband to give birth and Arabic women often bring their mother-in-law, which is really uncommon for German women. […]and there is for example that the baby has to be washed immediately.”* (Midwife, 28 years old)

### The Use of and Access to Maternity Care for Immigrant Women

Care for pregnant women and new mothers is defined in Germany in the guidelines for maternity care (*Mutterschaftsrichtlinien*) [22]. Women there are seen by their gynecologist, who has private ambulatory often only one-doctor-policlinics, or alternatively/or alternatingly their midwife—according to the woman’s choice—in increasing frequency. In the first months, the visits are monthly up to every other day after the due date. There are preparation courses for the birth as well as pregnancy sports classes (e.g. prenatal yoga) partially refunded by the insurance companies. During the first 12 weeks postpartum each woman is entitled to up to 26 home visits by a midwife to help during breastfeeding and childcare.

The participants talked about factors which influence the health care situation of immigrant women in Berlin. From their point of view, a low German language proficiency was a main cause for limited use of maternity care. Also, being already pregnant upon arrival in Germany or conceiving shortly thereafter were described as difficult conditions. According to the participants, some immigrant women tended to visit the doctor less frequently and/or wait until later in the pregnancy for the first antenatal care appointment or allow their pregnancy to go fully unmonitored.

*“(…) there are women with a history of migration, who are well informed, who regularly go to the gynecologist, who have health insurance, who maybe also speak moderate German or at least some German or always bring a translator, those are women of whom I think that they receive good antenatal care and then there are of course women who do not have a health insurance card, who do not find a doctor who speaks their language, who are therefore less well cared for in terms of the antenatal care they receive. Well, I think it is difficult to generalize, to say women or patients with a history of migration you cannot say it that way, it is dependent on which group of women with a history of migration is meant.”* (Physician, 29 years old)

Especially having a precarious legal residence status and not having health insurance was considered to have a significant influence on access to care. According to the participants, uninsured immigrant women do not receive the same standard of care. Most notably, these women were excluded from screening tests for birth defects.

*“(…) for women that do not have health insurance there is antenatal care at the health offices, and it is a only standard care, yet a quite a lot is missing that other women can purchase on top, like streptococcus tests or advanced ultrasounds, in these aspects they are less well served/cared for.”* (Midwife, 55 years old)

### Perceived Resources of Immigrant Women

#### Family Support

Many participants suggested that the broad family support structure evident among many immigrant populations partially compensated for immigrant women’s underuse of available structural support services (e.g. birth preparation classes). Furthermore, some participants described the important and supportive role of the family motivating and giving a helping hand during and after birth, in contrast to the non-immigrant women’s families. Many of the interview partners recognized strong family ties as a valuable postnatal support structure and by some as an even better alternative to the German postpartum-midwifery system. Reasons mentioned by the participants were that family members could be available all the time and that they speak the women’s language.

*“…For them, it is good. Yes, in any case, because they are their main contact persons during pregnancy, birth, the breastfeeding period, and childbed and well… That is totally fine, because they do a good job. Thus, also valuable the work of mother and grandmother (laughs). I would wish for this for some German women as well, that there are mothers who are a little bit more dominant and just give their daughters a hand.”* (Midwife, 31 years old)

Many interviewees saw the support immigrant women receive through their social network during labor and birth as a big resource:

*“…but they really support the women. Well, they don’t just sit there, they support, they motivate, they see this positively… much better than the Germans… and they get them over the mountain, and this mountain may be a lack of motivation but they get the women over it….. The Germans just come with their husband, who mostly doesn’t know what he should do.”* (Midwife, 53 years old)

Some participants also raised the point that external support as established in the German health care system might not be accepted by immigrant families. For example, a physician phrased it this way:

*“…do the women want that at all, no, because they have different standards, or wish for something different and in some regions of the world it is not so common that we have a midwife for postpartum care, what we nearly see as a precondition or at least standard already. And I think for a lot of people it is mostly the family that cares for these things and the broader family network and it is not wanted that someone external comes and intrudes and gives advice.”* (Physician, 29 years old)

Also a midwife had a similar impression:

*“ It depends, I have the feeling that midwifery support it not desired. I ask when they come to register ‘Do you have a midwife coming to your home’ and then they say ‘ehm, no, either I don’t want it or I don’t need it’. And often they have a lot of family at home that helps. Grandparents, partners, friends of the family […] well maybe it might be insufficient information. I tell them of course that it is for free, that the insurance covers the home visits of a midwife. Often, I have a feeling they are afraid and that they get the feeling of being controlled, although it is meant as a support. I have often heard ’No, I don’t want a midwife, they interfere with everything.’ I get a little sad, when I hear this, because in the end it is only an offer, which one can make use of.”* (Midwife, 26 years old)

Other participants see the lack of information for immigrant women as the main factor for lower use of institutionalized postnatal support. It was generally perceived that immigrant women were less likely to have a midwife in the postpartum period due to a lack of information about this support structure, a language barrier, or skepticism on behalf of the immigrant women towards an external (non-family member) professional assistance. In this area, some participants voiced their concern about immigrant women missing necessary professional assistance and, therefore, called for greater integration of these women into the postnatal care system.

*“Very often they don’t know what postpartum care means and when you ask them ‘Would you like the service of a midwife?’ ‘No, I don’t want that’ Because they don’t know that it is possible and that we are not biting and that we only want to help and that we do not cooperate with the child welfare office. I think there is still a lot of fear of contact and ignorance.”* (Midwife, 24 years old)

There were also respondents raising the issue that immigrants without broader family members present might be particularly vulnerable.

*“Culturally in the traditional postpartum period a lot of support is given by family members. And that is not always the case, because they sometimes don’t have family here, then there are big problems.”* (Midwife, 42 years old)

Some interviewees highlighted that the family support given might not necessarily be the best:

*“then you can only hope that the right social network, which they often do have, comes into play and then maybe the mother gives them some helpful advice, but, one doesn’t know, one doesn’t know if the advice is good or not [laughs]”* (physician, 28 years old)

#### “Natural Take on Giving Birth”

By the participants immigrant women were often perceived to tend towards a more natural approach to childbearing.

*“I believe, that in a lot of cultures childbearing is part of everyday life and part of family and of being and I think in Germany it is being hyped. There are less children being born relatively in Germany, that is always seen as something special, while in other cultures it is just normal, it is nothing special, but it is somewhat different. More natural.”* (Physician, 36 years old)

*“…no, there is a lot with gestures, and I would say they do a lot intuitively, well, they really still know how to give birth to a child, they know how to work with their bodies, I always like that a lot.”* (Midwife, 53 years old)

*“We are already very medicalized in our society. I mean there is actually no woman without an advanced ultrasound diagnostic during pregnancy, although this is often not medically indicated. We are super cautious and that is different in other cultures, I have the impression, that they don’t regard it as necessary as long as everything feels natural”* (physician, 31 years old)

Some respondents also link the social network and more “natural take on giving birth” to better coping mechanisms with pain during labor and birth:

*“Because they often have a better feeling of their body and because they often, when they live in a big family, are totally differently confronted with the process of giving birth. Because they have three, four, five siblings, the sister already has children, the wives of the brother have children and they are with them… they see pregnant women differently. The pregnant woman is not courted as much, but they know already much more about giving birth, they talk more – the women amongst themselves – how is it going to be, how is it with the pain, what do I have to do, when you move, when you stay up longer birth will get easier. Thus, this exchange is better in bigger families, better than when someone in Germany studies and does not know many women who have children and maybe just the exchange with her mother and sister via telephone. All the other friends are far away from having children and she might be the first one in her circle of friends and then it is making her feel anxious.”* (Midwife, 55 years old)

### Lower Expectations of Immigrant Women

Immigrant women are perceived as being more modest in terms of their expectations from the health care system. Some of the participants contrasted some of the non-immigrant women who are perceived as very demanding with immigrant women who do not have particular expectations on giving birth:

*“…concerning wishes for giving birth, which we ask for when we take the history of the women, I always have the feeling that many immigrant women just look at me flabbergasted and ask “what kind of wishes should I have for a normal birth” and the non-immigrant patients, they are more the type that they inform themselves and read a lot and sometimes they are there with a long plan for labor and birth. I have never seen this from an immigrant woman that she sits down and writes two pages about what she wishes for and what not.”* (Midwife, 26 years old)

Other participants see the reasons for lower expectations among immigrant women in their personal history and education and subsequent expectations from state institutions.

One midwife phrased it the following way, stressing different claims from a state between immigrant and non-immigrant women:

*“I think that non-immigrant patients come with a feeling that they have the right to be medically cared for. It is a basic right that everyone claims. I think immigrant women don’t do that. Most of the immigrant women they are rather grateful.”* (Midwife, 42 years old)

Another participant phrased it less in terms of legal claims but rather in terms of experiences made in the country of origin:

“*I would add that women who emigrate from a country where health care is not as good, they are happy just to be in a country that has accepted them and where they receive maternity care, that they then have less expectations. And are more grateful that there is someone at all that does antenatal check-ups and obstetric care.*” (Midwife, 28 years old)

### Voices of Migrant Women Giving Birth

Based on our review of the free-text comments from the MFMCQ study (secondary data), there was a common theme of gratefulness expressed by the patients who used the opportunity to provide comments.

Some of the patients voiced gratefulness for the care they received in Germany, because in their opinion, it was better than in their country of origin.

*“In general, I am very grateful to the Charité and all specialists that worked with me and my baby. We have a problem that could not be treated so well in Russia. And here we get the best treatment and support.”* (Mother from Russia, 28 years old)

Another woman from Slovakia also voiced that she values the pregnancy care in Germany very much, especially due to different standards of care in both countries:

*“In Slovakia, a lot of the offers that are in the German health system do not exist, thus I am very satisfied in Germany.”* (Mother from Slovakia, 27 years old)

Gratefulness was expressed also by a mother from Lebanon, although she also voiced the opinion that it is problematic that translators are not always available, not even for English:

*“Really happy that we came in this country. I am very thankful for the opportunity that I got. I respect everything I got - languages, food, places, people and everything. Yet I am wishing/hoping that places make sure they have an English translator. I guess it will help a lot and make the interaction very easy for those of us who can't understand their native languages. That's all. Thank you.”* (Mother from Lebanon, 30 years old)

## Discussion

Studying immigrant health is sometimes difficult due to the inconsistent indicators of migration and often no systematic collection of migration-specific data [23]. During this study, the health care professionals were asked themselves how they identified an “immigrant woman” or a “woman with migration experience”. The conclusion from the data we collected was that sometimes women were already categorized by their names before any/first personal contact. Within personal contact, especially women perceived as “different” were categorized as immigrants. A complex social world can be dealt with better by categorization and generalization, but stereotyping can also bear the risk of missing the individual’s needs[6]. This topic was not investigated further in the interviews but would be an interesting topic for further studies.

Studies on maternity care for immigrants often focus on discrepancies in care. Barriers to access for immigrants [2, 24, 25] or difficulties resulting from language barrier [26] are some examples. The study by Kasper regarding obstetric care for refugee women in Germany also mainly underlines the difficulties of refugee women in navigating the German health care system and overcoming a language barrier [10]. The analytic lens of CHC as used in this study can highlight other aspects of processes happening in the health care system. Immigrants might be excluded in some ways, but they might also have resources, compensating—at least partially—for these deficits. In the literature, CHC has been used to highlight how knowledge of the dominant culture facilitates integration into the institutionalized health care system [15]. In combination with critical race theory, CHC has also been used to show how alternative structures might be established to mitigate effects of exclusion from the institutionalized health care system [11].

Our research gives some insights into these processes. In general, the impression of health care staff in obstetric care is that immigrant women start later with antenatal care, and that especially with precarious immigration status and no health insurance the care is of lower standard. This could be shown for Canada for example [27]. There are studies from Berlin that support this impression for Germany as well. Especially women with low German proficiency, lower income, and no health insurance start antenatal care later and have fewer visits with an obstetrician during their pregnancy [4, 24, 28]. Especially, the restricted access to care for asylum seekers during the first 15 months in Germany has been criticized on an international level resulting in a low rating for Germany in the MIPEX health index [29].

In our interview material, we found various aspects that show alternative resources of immigrants. Their CHC leads to alternative structures that mitigate lower uptake of maternity care services. Our respondents, for example, often voiced the impression that the family support is so much higher among immigrants and this compensates for lower access to postnatal midwifery care. For example, the topic of breastfeeding has been linked to postnatal midwife support. A study from Canada highlights that predictors for breastfeeding duration differ between immigrant women and non-immigrant women in Canada [30]. This suggests that also in Germany, postpartum support for immigrant women encouraging breastfeeding needs to be tailored differently to immigrant women than non-immigrant women. Data on predictors for breastfeeding duration in Germany does not yet include immigration status [31]. In the analysis of the MFMCQ data, we found that less immigrant women (71%) compared to non-immigrant women (94%) know about the opportunity of postpartum care by a midwife coming for home visits. Among the women who knew about the services, 22% of immigrant women decided they did not want a midwife compared to 14% among non-immigrant women {Seidel, 2019 #464}. Our speculation is that better family support among immigrant women leads to a reduced need for institutionalized midwifery care. It would be interesting and important to investigate how family support among immigrant women correlates with breastfeeding rates and breastfeeding duration among these women.

Immigrant women are seen as having a more “natural take on giving birth” and thus are perceived to be more satisfied with the care they receive during labor and birth. Research arguing that the medicalization of giving birth in many western countries is alienating women from the natural process of giving birth ties nicely into this finding [32]. Furthermore, some respondents also link the family support and the “natural take on giving birth” to less need of pain medication during labor and birth. Moreover, the respondents had the impression that immigrant women had lower expectations. This might lead to less misled expectations and potentially a better birth experience. Listening to the immigrant women’s comments gave another clue as to why immigrant women seem to be so satisfied with the care they receive in Germany. Some compared the care in Germany to the care they would have received in their country of origin. They found the standard of care higher than in their country of origin, which supposedly led to a higher degree of satisfaction. This aspect might also explain why satisfaction with care among immigrant women is high although a potential language barrier is not always dealt with in the best way possible. In our quantitative analysis of immigrant women’s satisfaction with pregnancy care, we also found high rates of satisfaction among immigrant women {Gürbüz, 2019 #399}.

All of these aspects may be regarded as resources, but from a public health perspective we have to evaluate if this kind of “parallel structure” in maternal care is adequate. We know that the perinatal outcome for immigrant women in Berlin is not different from non-immigrant women [33]. This is encouraging. But do we know if there is postnatal care from a family member (e.g. grandmother, sister) or friend available and if it is equally safe as an institutionalized support by a trained midwife?

An intervention study in the UK shows that postnatal care by a midwife resulted in better mental health measures of the women in the intervention group [34]. There are no studies investigating the detection rate of postpartum depression by grandmothers in comparison. Furthermore, there are some traditional cultural practices—one extreme being female circumcision for example—that are potentially harmful to children [35]. A study in Turkey revealed high prevalence of potentially harmful cultural practices: swaddling (81%), dressing the baby with a sand-filled nappy (“holluk”) (35%), and bathing the baby in saltwater (40%) [36]. A professional midwife could detect such practices and prevent harm. A study in the Netherlands shows that the postnatal care of midwives was better accepted if cultural traditions were respected [37]. Future interventions regarding midwifery care for immigrant women should build on this knowledge.

Furthermore, there could be detected a certain “culturalization”, i.e. the respondents spoke in “we” and “them” thereby forming a perpetuating stereotypes. The “more natural” take on giving birth seen with immigrant women might be mostly in demarcation to a certain type of non-immigrant women giving birth that the respective respondent has problems with relating to.

The perception that pain is better compensated during labor and birth when the woman giving birth receives support by female family members must be critically analyzed. It is known that pain is differently expressed between cultural groups and this can inhibit the detection of pain by health professionals [38]. A disputed example of coping with pain during labor and birth is the lower rate of epidural anesthesia (EDA) among immigrants. A lower rate of EDA during labor and birth among immigrant women has been shown in various settings [39–41], but few authors give any reasons for this discrepancy. A qualitative study by Petruschke et al. among Turkish pregnant women in Berlin, being interviewed before giving birth, stresses mostly the “demand”-driven argumentation that immigrant women desire less often an EDA [42]. David et al. suspect rather that the “supply” to immigrant women in case of language barrier is limited [39]. A closer look on data regarding the lower rates of EDA among immigrant women from our study group also highlights the potential family dynamics within immigrant families where some family members might actively discourage or even inhibit a woman’s access to EDA [43].

The participants perceived immigrant women as having a more “natural take on giving birth” and lower expectations for care during labor and birth. There are numerous factors influencing the birth experience. A discrepancy between expected and actual experiences can lead to a negative birth experience [44, 45]. Thus, it might be a resource that immigrant women have less unmet expectations. None the less, the aspiration of a health care system should be equal care for all. Lower expectations should not lead to acceptance of substandard care.

### Strengths and limitations

To our knowledge, this study has been the first of its kind conducted in Germany. The study was performed in a convenience sample based on voluntary participation. Therefore, a selection bias must be considered as people already interested in the topic might have preferably taken part. The results only represent the views of the health care staff. The women’s perspectives obtained in this research project have a selection bias. It is a possibility that only women who were very satisfied made additional comments. Therefore, in this respect, our study should be regarded as hypothesis-generating. Further in-depth studies focusing on immigrant women themselves should follow to understand their respective CHC better.

The study does not explicitly differentiate between forced immigration and voluntary immigration. There might be differences regarding social resources in the country of destination depending on the reason for migrating. Qualitative research on immigrant women’s perspectives of the obstetric care they receive should follow to paint a full picture of the current situation. The study is only conducted in Berlin, the capital and biggest city of Germany, the results might not all be transferrable to other parts of Germany. Furthermore, the study generalizes very much between “immigrant women” and “non-immigrant” women. Different cultural groups might have very different traditions and each individual of a particular culture might behave differently.

## Conclusion

The theoretic lens of CHC helps to see the resources of immigrant women. Family support is essential for home support of mothers before, during, and after giving birth. The perception that immigrant women have a more “natural take on giving birth” is an impression by many respondents that should be further investigated through qualitative studies with immigrant women themselves. Until more knowledge is obtained, health care professionals should beware of making early conclusions. This perception could be misleading due to different cultural ways of expressing pain and dynamics within immigrant families.

In any case, equal standards of care are an important prerequisite for good public health. CHC mitigates certain aspects of exclusion from the regular health care system. This is important to notice, but also to act on. Hence, the existing resources of immigrants should not be accepted as an alternative, but any public health intervention should be built on existing resources and potentially integrate them.

## Supplementary Information

ESM 1 (DOCX 14 kb)
