# Supplementary material for: Equal Alternatives or Lower Standards for Immigrant Women—Analyzing Obstetric Care for Immigrant Women in Berlin Within the Framework of Cultural Health Capital
Source: J Racial Ethn Health Disparities. 2023 Aug 15;11(5):2689–98. doi: 10.1007/s40615-023-01732-0 (PMC11480289; doi:10.1007/s40615-023-01732-0)
Supplement: Supplementary file 1 — (DOCX 14 kb) [file 40615_2023_1732_MOESM1_ESM.docx]

Appendix 1: Interview guideline (English version)

Interview-Code: Date:

Hospital:

Time beginning:

Time ending:

Interview “the situation of obstetric care for immigrant women in Berlin through the eyes of the health care workers“.

1. Please tell me about a recent situation with an immigrant women in the delivery ward that stayed in your memory.

2. How often do you have contact with immigrant women in your daily routine?

• Which different types of groups of immigrant women would you differentiate?

• How do you recognize if someone has a migration experience?

3. Do you see similarities and differences concerning prenatal care for immigrant and non-immigrant women? If yes, which ones?

• Do immigrant and non-immigrant women according to your experience have the same expectations regarding the care during delivery?

• Which differences and similarities do you see?

4. What do you think the reasons are for different expectations?

If yes: Regarding the differences you mentioned, according to your opinion which factors are important? (cultural, social, language/communicative)

5. How do you evaluate the postpartal care for immigrant and non-immigrant women in Berlin? / Do you have the feeling; immigrant women are informed about respective options?

6. Do you have the impression that since the new influx of refugees that the care for immigrant women in Berlin has changed?

7. Are you overall satisfied with the current situation of care for women?

What would you wish from non-immigrant women to make your daily work more satisfying? What would you wish from immigrant women to make your daily work more satisfying?

8. What would you wish from the clinic to make care for pregnant women easier?

9. What resources does the clinic offer to help in a situation of a language barrier?

10. Are you making use of these resources regularly?

11. How do you feel if you are taking care of a woman during labor and birth whose language you do not speak?

- Regarding this discomfort you are voicing, what is your biggest fear?

- What obstacles do you see/have in your daily work?

12. The Berlin perinatal study has shown that pregnant women from Turkey and Lebanon have lower c-section rates than non-immigrant women. Why do you think this is the case?

13. The Berlin perinatal study has shown lower rates of epidural anesthesia among women from Turkey and Lebanon compared to non-immigrant women. Why do you think this is the case?

14. Who do you think is responsible for overcoming a language barrier?

15. Would you say that your clinic has begun with measures for intercultural opening? Which are these? Are you regarding these as helpful in caring for immigrant women?

16. Did you ever take part in an event or a training on intercultural communication or intercultural competence?

a) If no: Do you wish for a training on how to improve care for immigrants? Which kind?

b) If yes: further questions: which kind? Was it voluntary or during work time? Was it helpful?

17. Do you wish for other support in giving care for immigrant women?

18. Do you want to add anything?
